# Implementing supportive exercise interventions in the colorectal cancer care pathway: a process evaluation of the PREPARE-ABC randomised controlled trial

**DOI:** 10.1186/s12885-021-08880-8

**Published:** 2021-10-23

**Authors:** Jamie Murdoch, Anna Varley, Jane McCulloch, Megan Jones, Laura B. Thomas, Allan Clark, Susan Stirling, David Turner, Ann Marie Swart, Kerry Dresser, Gregory Howard, John Saxton, James Hernon

**Affiliations:** 1 Department of Population Health Sciences, King’s College London, London, SE1 9NH UK; 2 School of Health Sciences, University of East Anglia, Norwich, NR4 7TJ UK; 3 Norwich Clinical Trials Unit, Norwich Medical School, Faculty of Medicine and Health Sciences, University of East Anglia, Norwich, UK; 4 School of Sport and Exercise Sciences, Liverpool John Moores University, Tom Reilly Building, Byrom Street, Liverpool, L3 3AF UK; 5 Department of Sport, Health and Exercise Science, University of Hull, Hull, HU6 7RX UK; 6 Norfolk and Norwich University Hospitals NHS Foundation Trust, Colney Lane, Norwich, NR4 7UY UK

**Keywords:** Colorectal cancer, Surgery, Exercise prehabilitation, Rehabilitation, Process evaluation

## Abstract

**Background:**

A colorectal resection is standard treatment for patients with colorectal cancer (CRC). However, the procedure results in significant post-operative mortality and reduced quality of life. Maximising pre-operative cardiopulmonary fitness could improve post-surgical outcomes. PREPARE-ABC is a multi-centre, three-armed, randomised controlled trial investigating the effects of exercise interventions, with motivational support on short and longer-term recovery outcomes in CRC patients undergoing major lower-gastrointestinal surgery. The trial included an internal pilot phase with parallel process evaluation. The aim of the process evaluation was to optimise intervention implementation for the main trial.

**Methods:**

Mixed methods process evaluation conducted in 14 UK hospitals between November 2016 and March 2018. Data included a site profile questionnaire and telephone scoping interview with hospital staff, 34 qualitative observations of standard care and 14 observations of intervention delivery, 13 semi-structured interviews with healthcare professionals (HCPs) and 28 semi-structured interviews with patients. Data analysis focused on describing intervention delivery within each arm, assessing fidelity, acceptability and how variation in delivery was linked to contextual characteristics.

**Results:**

Standard care exercise advice was typically limited to maintaining current activity levels, and with lead-in time to surgery affecting whether any exercise advice was provided. Variation in HCP capacity affected the ability of colorectal units to deploy staff to deliver the intervention. Patients’ exercise history and motivation prior to surgery influenced HCP perceptions and delivery of the motivational components. Observations indicated a high level of fidelity to delivery of the exercise interventions. All but one of the 28 interviewed patients reported increasing exercise levels as a result of receiving the intervention, with most finding them motivational and greatly valuing the enhanced level of social support (versus standard care) provided by staff.

**Conclusion:**

Hospital-supervised and home-based exercise interventions were highly acceptable for most patients undergoing surgery for CRC. Delivery of pre- and post-operative exercise within the CRC care pathway is feasible but systematic planning of capacity and resources is required to optimise implementation.

**Supplementary Information:**

The online version contains supplementary material available at 10.1186/s12885-021-08880-8.

## Background

Colorectal cancer (CRC) is the fourth commonest cancer in the UK, with 40,000 patients diagnosed per year [1]. The current standard, and best-proven treatment for this patient group is a surgical resection with approximately 25,000 patients in the UK undergoing a major abdominal resection each year. Colorectal resection offers the best chance of cancer survival, with a 90-day post-operative mortality of 3% [2]. Considerable research has focussed on improving post-surgical outcomes. Studies have reported an association between cardiopulmonary fitness and post-operative recovery outcomes in cancer patients, including those undergoing colorectal resection [3–5]. This suggests that maximising pre-operative cardiopulmonary fitness could improve post-surgical outcomes. However, delivery within cancer care pathways and adoption by patients presents challenges within resource-constrained healthcare systems. Variability of implementation across different NHS contexts, and where the interventions are being delivered by multiple individuals, provides an additional layer of complexity. Process evaluation is therefore critical for understanding how exercise interventions can be effectively implemented within hospital settings, by assessing the extent to which they are implemented as intended, identifying reasons for variation in delivery and specifying the circumstances under which the intervention is likely to succeed.

The process evaluation reported here formed part of the pilot phase of PREPARE-ABC, a multi-centred, three-armed, randomised controlled trial investigating the effects of exercise interventions, with motivational support on short and longer-term recovery outcomes in CRC patients undergoing major lower-gastrointestinal surgery. The trial included an internal pilot phase to allow an assessment of stop/go criteria for progression to a full trial. The aim of the process evaluation was to identify recommendations for improving intervention delivery within the main trial. A full protocol of the trial is reported elsewhere [6]. Here, we describe the organisational context of the hospital sites delivering the PREPARE-ABC exercise interventions, the nature of standard care, how staff organised intervention delivery within their particular care setting and how patients experienced the intervention.

## Research design and methods

### Overview of the PREPARE-ABC RCT

Trial participants were aged 18 years or over and were awaiting a curative elective colorectal resection for cancer (laparoscopic or open) [6]. They were randomised to either (i) hospital-supervised exercise; (ii) home-supported exercise; or (iii) treatment as usual.

The delivery of the exercise interventions was underpinned by self-determination theory (SDT) [7] which aims to develop an individual’s motivation towards sustainable behaviour change, achieved by satisfaction of the three basic psychological needs – to feel autonomous, competent and related (having a sense of belonging). Participants randomised to one of the exercise interventions attended an initial 45-min counselling session at their treating hospital to initiate this process of self-determination, followed by face-to-face exercise sessions in the hospital-supervised arm, and motivational telephone calls in the home-supported arm.

There were pre- and post-operative phases to intervention delivery and participants randomised to one of the exercise interventions were asked to follow a structured exercise programme during both of these phases. After the initial counselling session, patients in the hospital-supervised arm were asked to attend up to three aerobic interval exercise sessions per week on a cycle ergometer over the 3–4 weeks prior to surgery, plus two home-based resistance exercise sessions per week using resistance bands. Patients randomised to the home-supported arm were asked to comply with current public health physical activity guidelines [8] by engaging in at least 150 min of moderate-vigorous intensity aerobic exercise per week (e.g., brisk walking/jogging/cycling/ swimming), as well as completing two weekly sessions of resistance exercise using resistance bands. Six weeks after surgery, patients in the hospital-supervised arm were offered monthly “booster” supervised exercise sessions at the hospital, whilst those in the home-supported arm received monthly 15 min telephone counselling calls to support continued engagement in their programme. In the post-operative phase, both groups were encouraged to comply with current public health physical activity guidelines [8]. Patients randomised to the control arm received standard care, which did not include formalised pre- and post-operative support for undertaking exercise. The trial is listed in the ISRCTN registry (ISRCTN82233115; date of registration: 07/07/2016).

### Process evaluation design and methods

MRC guidance on process evaluation [9] was used to design a mixed methods process evaluation during the pilot phase of PREPARE-ABC. The process evaluation aimed to: 1) describe the wider context into which the interventions were introduced and the nature of standard care; 2) assess how the exercise interventions were delivered and fidelity to the intervention protocol; 3) assess patients’ and staff experiences and acceptability of the interventions; and 4) assess any variation in non-receipt of the interventions in the control arm and any sources of contamination. Here, we focus on the first three of these objectives, in order to make recommendations on how to implement the exercise interventions in hospitals more widely. Methods and results have been described using the Consolidated Criteria for Reporting Qualitative Research (COREQ) [10].

Data were collected from the first cohort of 14 hospitals participating in the trial, using a site profile questionnaire to collect quantitative data on characteristics (Supplementary File 1) of each colorectal unit; a telephone scoping interview with hospital staff collecting quantitative and qualitative data on standard care patient pathways (Supplementary File 2); qualitative researcher observations of standard care consultations and intervention sessions; and qualitative semi-structured interviews with staff and patients. Data were collected between November 2016 and March 2018.

### Participants and recruitment

Participants were aged 18 years or over and were undergoing surgery for CRC. HCPs were those with a role in providing CRC care, including colorectal nurse specialists (CNS), consultant colorectal surgeons, physiotherapists, research practitioners, cardiopulmonary exercise test (CPET) technicians and exercise practitioners. There were two streams of patient and staff recruitment for the process evaluation:

1. To evaluate the wider context into which the interventions were introduced, a sample of patients were recruited before the internal pilot trial commenced. Patients requiring surgery for colorectal cancer were approached by staff for written consent to allow a researcher to observe their pre- and post-operative consultations received as standard care. Research staff approached members of staff to advise them that participation was optional and to obtain consent where appropriate. Patients and accompanying adults recruited via this stream were not recruited to the trial.
2. Patients and staff who had already provided consent to participate in the trial were approached to participate in an observation, or interview (3–6 months following surgery), about their experience of the intervention or standard care. Participants were provided with additional written information, informed that participation was optional and their informed consent secured.

Delays in site set-up and recruitment, and logistical challenges of observing exercise intervention sessions necessitated design changes to the sampling strategy. Instead of purposively sampling patients and staff across all 14 hospitals, we obtained a convenience sample within eight of the 14 participating sites, comprising: 16 patients and 23 clinicians across four hospitals who had at least one pre- and/or post-operative standard care consultation observed; eight patients and five clinicians across five hospitals who had at least one intervention session observed; 13 clinicians across seven hospitals who were interviewed about delivering the intervention; and 28 patients across seven hospitals who were interviewed about their experience of the intervention or standard care (hospital-supervised arm = 10; home-supported arm = 13; standard care = 5). Table 1 provides a breakdown of characteristics of patient participants randomised to each trial arm. Comparable data for the 16 patients recruited to standard care observations (recruitment stream 1) were unavailable.

**Table 1:** Characteristics of randomised patients participating in process evaluation

Treatment arm	Gender	Ethnicity	Age	Employment Status
Hospital-Supervised Exercise	Male (*n* = 9) Female (*n* = 1)	White British (*n* = 9) North African (*n* = 1)	Range 39–79 years Mean 65 years	Employed (*n* = 4) Retired (*n* = 5) Unemployed (*n* = 1)
Home-Supported Exercise^a^	Male (*n* = 8) Female (*n* = 6)	White British (*n* = 14)	Range 59–85 years Mean 71 years	Employed (*n* = 2) Self-employed (*n* = 1) Retired (*n* = 11)
Treatment as Usual	Male (*n* = 4) Female (*n* = 1)	White British (*n* = 5)	Range 68–80 years Mean 73 years	Employed (*n* = 2) Retired (*n* = 3)

^a^Includes 13 patients who were interviewed but not observed, and one patient who was observed but not interviewed

### Data collection

**Fig. 1:**
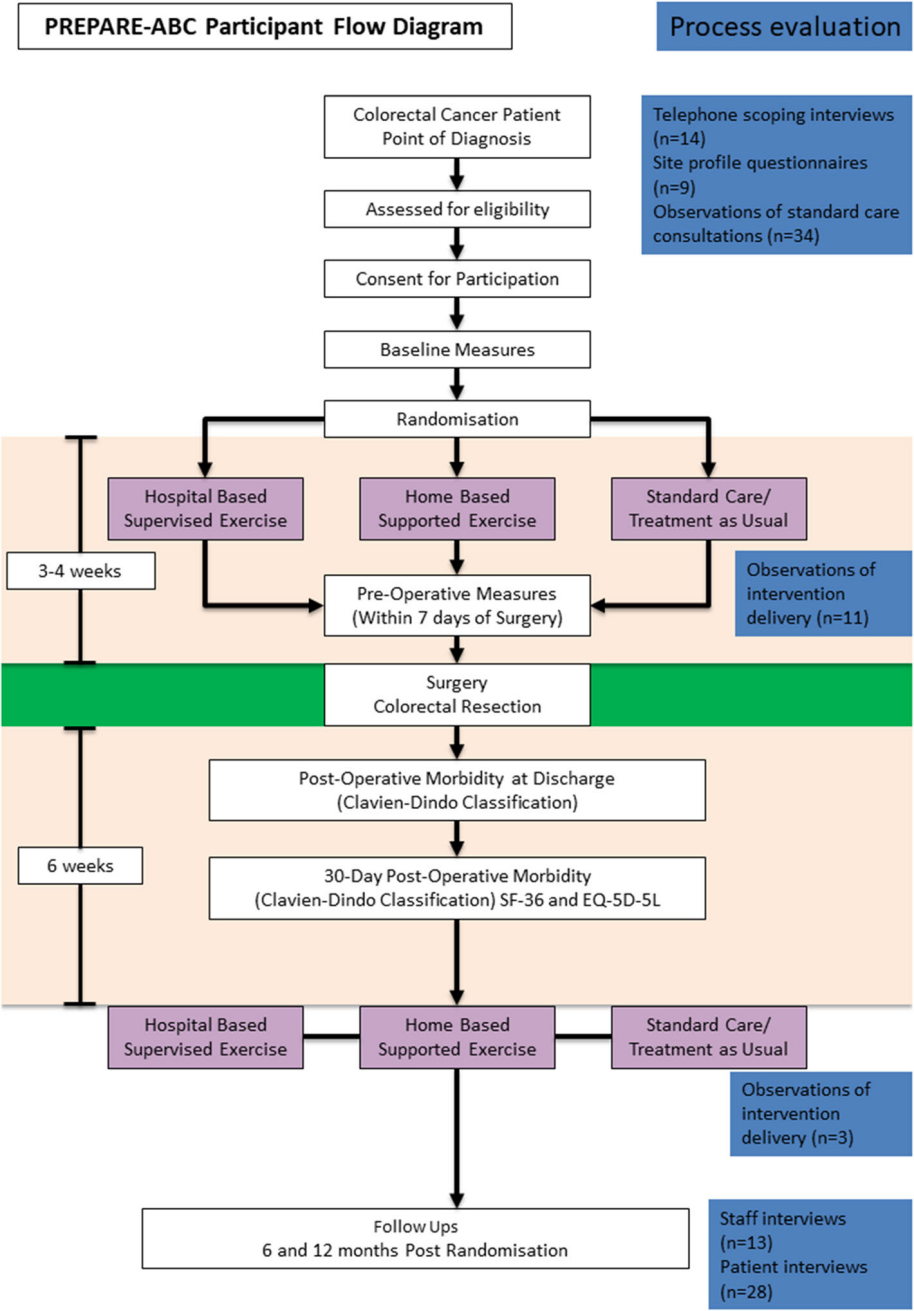
Process evaluation components alongside trial participant flow diagram

Figure 1 provides details of each component of the process evaluation set alongside a participant flow diagram for the main trial. Telephone scoping interviews with staff were conducted at all 14 hospitals, nine of whom completed and returned the site profile questionnaires. A total of 34 consultations of standard care were observed across the patient journey, totalling 13 ½ hours of observation time, including pre-operative consultations for diagnosis, pre-operative assessments, CPET appointments and post-operative consultations with a surgeon or CNS. These observations were designed to deepen our understanding of the wider context into which the intervention was being implemented, identify differences in practice between the trial interventions and standard care, including insight into what specific information was provided regarding exercise, and to establish what changes would be required within sites to deliver the exercise interventions.

A total of 14 observations of intervention delivery were carried out across five sites, totalling 13 h of observation time. Eight patients were observed. One patient was observed on five occasions and one on three occasions. The remaining patients were observed once. A range of professionals were observed delivering the counselling and exercise sessions, including two physiotherapists, one research practitioner, one exercise practitioner and one colorectal consultant. A semi-structured guide was used for all observations, requiring the researcher to take fieldnotes of how different consultation activities were structured, delivered by staff and responded to by patients.

A semi-structured interview schedule was used to guide the 13 staff interviews which aimed to cover experiences and perceptions of delivering the different components of the counselling and exercise sessions, organisational and individual barriers and facilitators of delivery, and views of how patients responded to motivational and exercise elements of the intervention. Staff included nine research practitioners, three physiotherapists and one exercise practitioner. The 28 patient interviews were semi-structured around a topic guide designed to explore the patient’s experience of participating in the trial, their experiences of being diagnosed with and receiving treatment for colorectal cancer, previous habits, motivation for exercise and behaviour change, following participation in the study. Patients were typically interviewed at home, 3–6 months following surgery. Data were primarily collected by AV (Social Scientist) supported by JMc (Colorectal Research Nurse) and JM (Social Scientist). AV, JMc and JM had no involvement in the running of the main PREPARE-ABC trial or delivery of the intervention. All procedures for data collection, analysis and write-up of the process evaluation was conducted independently from delivery of the main trial.

### Data analysis

Audio recordings of qualitative interviews were transcribed verbatim and anonymised. A deductive coding framework was then used to structure thematic analysis [11] of transcripts (Supplementary File 3), an appropriate approach where specific questions about intervention components were identified a priori, in this case how the different elements of the counselling and exercise sessions were delivered by staff and received by patients. Transcripts were also analysed inductively to allow unanticipated themes to be identified, using techniques of constant comparison [12] to identify themes relevant to delivery, fidelity and acceptability, including searching for disconfirming cases and any themes not captured by the coding framework. Qualitative fieldnotes from observations were analysed to provide a description of standard care, or intervention delivery and fidelity, and for how variation in delivery was linked to relevant contextual features within sites.

To interpret intervention fidelity, we examined how the sequence, structure and delivery of intervention sessions corresponded to delivery set out in the intervention manual, documenting a description of how each activity was carried out, noting any deviations from the protocol. We then triangulated these descriptions with perspectives of delivery reported in interviews in order to identify evidence of variation from our interpretation of fidelity and how delivery needed to adapt according to the patient and circumstances of delivery [9]. Conversely, to interpret acceptability of the intervention we analysed interview transcripts to look for points of convergence and divergence between participants regarding each intervention component. We then triangulated our interpretations (manifested as themes) with observational fieldnotes to assess the extent to which views of acceptability were evident in how the practitioner and patient negotiated each activity within the intervention session, for example looking for evidence that patients were engaged whilst carrying out aerobic activity on the cycle ergometer.

Analysis was undertaken by AV and JMc, whose respective backgrounds provided points of convergence and divergence in interpretation. Disagreements were resolved through validating within and between interview transcripts and observational fieldnotes, and where possible with participants. Quantitative data collected from site profile questionnaires and telephone scoping interviews were analysed to provide descriptive statistics of each colorectal unit. All data were triangulated to generate a ‘thick description’ [13] of how each arm of the study was delivered, maintained and experienced by staff and patients. The analysis of the interview and observational data was therefore iterative, with knowledge gained from observations of both standard care and intervention delivery used to strengthen the interview topic guide and provide additional insights during analysis.

### Ethical considerations

The East of England – Essex Research Ethics Committee (Reference: 16/EE/0190) approved the trial at all participating sites. Consent for the process evaluation was requested separately and after participants had consented to the main trial, with the exception of those participating in the evaluation of standard care prior to main trial recruitment. Written informed consent for interviews and observations was obtained from all participants. During the consent process it was made clear that the participant could decline to participate in the process evaluation at any time and for any reason, without affecting their future care or treatment.

## Results

The results report findings within three areas: 1) the wider organisational context of colorectal units and nature of standard care; 2) delivery and fidelity of the exercise interventions, comprising the initial counselling session, applicable to both interventions (hospital-based supervised exercise sessions and the home-based supported exercise components); and 3) acceptability of delivering both intervention arms within hospital settings. Illustrative quotes from qualitative data are included to represent key issues we identified regarding how intervention components were implemented, fidelity and acceptability to staff and patients.

### Wider organisational context of colorectal units and standard care

Table 2 provides characteristics of the nine out of 14 colorectal units which responded to the site profile questionnaire, demonstrating wide variation in the numbers of new colorectal cancer cases relative to the numbers of staff employed to provide care within each unit.

**Table 2:** Characteristics of pilot sites completing site profile questionnaire

Site	No. of new Colorectal Cancer cases in 2016	No. of Colorectal Resections in 2016	% Resection to total Colorectal Cancers	No. of Colorectal beds	No. of full-time equivalent (FTE) Colorectal Nurse Specialists	No. of Enhanced Recovery After Surgery (ERAS) Nurses (All Part time)	No. of FTE Colorectal Physiotherapists
**SITE 1**	510	325	64%	76	3	2	3
**SITE 2**	433	263	61%	37	2.5	0	1
**SITE 3**	406	287	71%	28	3	0	0
**SITE 4**	400	320	80%	28	4	0	2
**SITE 5**	326	177	54%	48	1.8	0	0
**SITE 6**	273	150	55%	29	0.8	0	0
**SITE 7**	237	166	70%	32	2.5	2	0
**SITE 8**	236	151	64%	55	3	0	0
**SITE 9**	200	180	90%^a^	29	1	0	4

^a^highest reported percentage of resections from Site 9 must be viewed with caution because it does not match the figure reported in the National Bowel Cancer Audit (NBOCA) Report Version 2 for 2016 [2]. This discrepancy may be due differential interpretation of data by sites and the categories in the NBOCA Report

Five sites did not employ a dedicated colorectal physiotherapist at the time of commencing the trial. Such variation in capacity presented challenges in the ability of some colorectal units to deploy staff to deliver the intervention, and the ability to secure rooms to counsel and exercise patients.

*It’s full on if we have a patient in the [supervised exercise] hospital based, could come in 3 times a week, or there could be nothing for a period of time, but then the next patient could appear and we are off again. So, managing the time is not that easy and always being available when you are needed and we have also issues around rooms to use and that kind of thing. Although we started with having something booked regularly, but then did not have a regular patient every week then we have had to forfeit that, and book rooms as and when, so that side of things has been more of a challenge than the actual delivery itself.* (Interview, Physiotherapist)

### Colorectal cancer care pathways and exercise advice

All 14 sites reported in their telephone scoping interview that they had an Enhanced Recovery After Surgery (ERAS) policy in place for colorectal patients covering a range of pre-, peri- and post-operative elements to prepare patients for surgery and enhance their recovery post-surgery. Only two sites reported employing an enhanced recovery practitioner or facilitator to implement the elements and none of the ERAS elements were related to exercise.

#### Pre-operative

Patients saw a range of professionals pre-operatively. Staff at all 14 sites reported it was standard care for patients to attend a diagnosis appointment with a consultant colorectal surgeon and see a CNS pre-operatively. In 12 out of 14 sites, patients also attended a pre-assessment clinic, which usually included an appointment with an anaesthetist. We identified only one site where patients saw a physiotherapist prior to surgery. The primary purpose of the pre-operative appointments was to inform patients of their diagnosis, and discuss their treatment plan and surgical options. The pre-operative sessions were also an opportunity for HCPs to advise patients to get themselves as medically fit as possible prior to surgery, referring patients back to their GP or a specialist to treat any underlying medical conditions. Fifty percent of sites stated that patients were routinely offered advice about exercise in the pre-operative phase. However, while it was emphasised that fitness was important for surgery outcomes, we found that patients were not encouraged to increase activity levels.

CNS: *“What keeps you busy?”*

Patient: *“Golf, snooker and TV”*

CNS suggests “*carrying on as normal.”*

(Observation, Pre-operative appointment, CNS)

Nine out of 14 sites reported that patients completed a CPET in order to assess their fitness prior to surgery. Typically, CPETs appeared to function purely as a mechanism for the anaesthetist to determine the patient’s fitness for surgery. An exception to this was a patient with a chronic lung condition:

Anaesthetist: *“The problem is, you only have two weeks until your operation. Can we get you fitter before surgery?... I worry about how I would get you off the ventilator and at the moment I don’t think you are fit enough for an operation. We could try an exercise programme but it is no guarantee- that’s a 10 week programme.”*

(Observation, Pre-operative appointment, Anaesthetist)

Lead-in time to surgery was clearly an important factor for determining whether pre-operative advice about exercise was provided:

*“If there is a long run up to surgery, exercise will get mentioned, however the window of time is short and so it is often not a priority to mention it*.” (Telephone interview, CNS)

#### Post-operative

Patients were routinely transferred to a high dependency unit for the first 24 h following surgery. At all sites, once patients were transferred to the ward, they were seen by a CNS at least once. At 13 out of 14 sites, patients were seen by a physiotherapist (including non-colorectal cancer physiotherapists) to provide support with mobility and respiratory health. The number of visits by the physiotherapist during the patient’s hospital stay ranged from daily to only one occasion and focussed on patient’s fitness for discharge.

In contrast to pre-operative advice, patients received more targeted guidance on the importance of activity following surgery: *“Both operations come with some kind of risks and it’s important that we go through those.”* The surgeon explains that there is a risk of post-operative bleeding and clots in the legs and lung, and to prevent that: *“we bully you a bit to get you up and moving as soon as possible after the operation.”* (Observation, Diagnosis appointment, Surgeon).

Clinical advice on the importance of activity was reinforced with leaflets detailing post-treatment rehabilitation courses tailored to the individual recovering from cancer treatment. However, advice was delivered as a general suggestion rather than a prescribed and systematic exercise regimen and there was no evidence of staff attempting to encourage exercise targets or support the patient’s motivation to exercise.

### Delivery and fidelity of exercise interventions within hospital settings

#### Initial counselling session

Staff emphasised the importance of the counselling session for motivating patients to exercise. An exercise practitioner with extensive experience of motivating inactive, unmotivated patients provided some insight into why he felt the counselling session was necessary.

*Just from experience, it’s vital because many people come in and look for excuse after excuse after excuse for not doing stuff … that’s the ones where motivational interviewing really is of prime importance as opposed to the person who says yeah, I want to get fitter...the people who are coming in are even struggling to walk up a flight of stairs and so on they’re the ones where you really have to try and engage and find ways around certain barriers that they have in their own minds* (Interview, Exercise Practitioner)

Motivating such patients required practitioners to deploy sophisticated communication skills as illustrated in the following interaction between a physiotherapist and patient:

Physiotherapist (Ph): *“Are you doing any activity at the moment?”*

Patient (Pt): *“Um, no, no not since I had to have my dog put down, no … I was walking with him.”*

Ph: *“But since then not much of anything?”*

Pt: *“No.”*

Ph: *“O.k. that’s fine. We just want to build you up a little bit”*

Pt: *“I must say that I’m devoted to my wheels. I hope to keep driving for a long time so you’ve got to drive every day.”*

Ph: *“Yeah, that’s right. We just have to add some walking into that as well otherwise we’ll fail the trial”* Ph says joking and patient laughs.

Ph: *“Start parking a bit further away.”*

(Observation, Exercise counselling session, Home Arm)

In contrast, staff reported that they struggled to implement the self-determination element of the counselling session when faced with a patient who was already motivated. This created difficulties for staff in attempting to follow the structure of the counselling session as set out in the training, whilst also attending to individual patient needs. Some staff continued to try to adhere rigidly to the intervention protocol whilst others were observed covering only certain elements whilst glossing over others.

*He was very difficult actually because there wasn’t really that much we could do to boost him up. He came in for the bike once but at home he was already rowing on his rowing machine, he had a weight machine, he was cycling every day. There wasn’t really that much more we could offer, he was going to the gym.* (Interview, Physiotherapist)

#### Supervised exercise sessions

As we report in the findings from the pilot trial [14], 57% patients in the hospital-supervised group attended ≥6 pre-operative sessions and 50% attended ≥5 monthly post-operative exercise “booster sessions.” A variety of HCPs were observed delivering exercise sessions according to the instructions set out in the manual, indicating a high level of implementation fidelity. Patients were instructed, monitored and timed in line with trial protocols.

*We strap them up with a polar monitor, we literally put them on the bike attached to a blood pressure machine and off they go and then they do five minutes and then a rest for 2.5, I’d have to look at the manual, that’s when I would look at my manual.* (Interview, Physiotherapist)

Variations in how the repetitions on the exercise bike were delivered were minimal between different sites and between HCPs within sites, where more than one person delivered the sessions. Where variation did occur it pertained to elements such as warm-up exercises and differences in the bikes used.

Chemotherapy, post-operative complications and an inability to sit on the bike seat due to discomfort following the operation, were the most common reasons for interruptions in sessions or delay in re-starting hospital supervised exercise sessions following surgery.

*… because I had had a targeted biopsy for the prostate it made it extremely painful to ride the appropriate exercise bike that was in the study. The one that they had in the lung function area was a lot more comfortable.* (Interview, Patient, Hospital Arm)

#### Home-based supported exercise

As we report elsewhere, in the home-supported group [14], 70% patients participated in ≥2 telephone support sessions in the pre-operative phase and 80% participated in ≥5 monthly telephone support “booster sessions.” In a similar way to patients in the hospital group, patients undertaking home-based exercise spoke positively about the impact of the study on their levels of exercise. All but one patient reported that they had increased their exercise levels as a result of participating in the trial. Patients supported their claims by citing numbers of steps walked, providing information regarding previous and current exercise levels and showing the interviewer their completed exercise diaries. One patient went from doing no exercise pre-trial to starting to do gentle walking, but typically patients carried on with current activities at a higher level of intensity, alongside taking up one or two new activities such as swimming, cycling or joining the gym.

*I’m pedalling and the rowing and strengthening exercises. You know on your thighs and your legs and pushing up and that sort of thing. So that’s what I’m doing, it’s only for half an hour twice a week and also swimming which (name of practitioner) will be really pleased about because he says I think you ought to swim!* (Interview, Patient, Home Arm)

The lack of ability to monitor home patients effectively was identified as being a challenge by staff in this arm. Many staff reported difficulties getting hold of patients to carry out the telephone calls:

*Well the first time I rang him, he was in the pub. The second time, he didn’t answer because he was in the pub and we’ve only managed three phone calls with him because the last time he was in the pub and didn’t ring us back so, mm.* (Interview, Physiotherapist)

### Acceptability of implementing the exercise interventions within hospital settings

Table 3 provides a breakdown of themes and sub-themes that we identified in patient and staff interviews which contributed to the assessment of acceptability of implementing both intervention arms within hospitals. Our data revealed how acceptability of the exercise interventions could not be isolated from the wider context of delivering the intervention within busy hospital settings. Themes of acceptability therefore represent this relationship, representing perspectives of intervention components and the ability to implement in everyday clinical practice.

**Table 3:** Themes and sub-themes identified in staff and patient interviews contributing to assessment of acceptability of implementation of intervention arms

	Themes	Sub-themes
**Supervised exercise arm**	Motivation	• Social exercise as mechanism for motivating patients • Counselling critical for inactive patients^a^ • Motivating patients requires sophisticated communication skills
	Social support	• Flexible, individualised support according to prior motivation and exercise levels • Understanding patient capacity
	Interruptions	• Chemotherapy • Post-operative complications • Inability to use bike seat
	Constraints within CRC care context	• Lead-in time to surgery limiting pre-operative exercise • Limited staff capacity • Limited space and availability of exercise bikes
**Home-based exercise**	Social support	• Regular contact on phone • Supportive, ongoing relationships as motivational • Advice on restarting exercise post-operatively • Unwanted pressure to increase activity • Difficult for HCPs to monitor progress
	Increased activity at home	• Higher intensity of current activities • Taking up new activities

^a^Patients in both arms received the pre-counselling session. This sub-theme therefore also applies to patients in the home-based arm

#### Supervised-exercise arm

Despite many patients reporting being active and having motivation to exercise prior to participating in the trial, there was evidence that patients experienced the exercise sessions as motivational. This became particularly apparent in the patient interviews when patients talked about why they were glad to have been randomised to receive the hospital-supervised arm.

*I’m not certain how it would have worked if I’d say had to do it at home because exercising in a solitary manner is difficult, which is why my cardio rehab classes are so much better. They’re a social exercise. Coming here to use the bike, again it’s more social and you are actually doing something with somebody.* (Interview, Patient, Hospital Arm)

The notion of “social exercise” was reiterated by patients and staff as a key mechanism for motivating patients, observable within this post-operative session where one such patient was supported to increase their level of resistance:

Research Practitioner (RP) asks if they can *“try another 0.5 kg”*

Pt: *“No, this is how I like it.”*

RP: *“We are doing it fairly light and we’d like to get you up to 13-15”* referring to the numbers on the Borg scale.

Pt: *“O.k. we’ll give it a go.”*

#### Home-based supported exercise

The majority of patients greatly valued the regular contact from staff providing post-operative telephone counselling calls, offering illustrations of how the practitioners built supportive, open relationships with patients that functioned to motivate and change the patient’s activity.

*He sounds as if he’s like a friend. He doesn’t make it sound as if he’s asking me personal questions. He just phones me up, hello “name of patient”, lovely to talk to you, how are you feeling? And I said feeling great (name of practitioner) and he said great, he said how’s your exercising going? And I’m honest with him about that. I said I’ve got a bike in the garage now. He said oh brilliant* (Interview, Patient, Home Arm)

However, two exceptions were patients who found the telephone calls to be challenging because they felt that they were already active enough and did not want to be pressured into doing any more activity. They felt strongly that they were being asked to do too much by the study team.

*Well I try to do it in the morning (the resistance bands) but (name of practitioner) suggested to do it in the afternoon. I says I’m 82-year-old, I’m not going in for the World Olympics or owt like that you know.* (Interview, Patient, Home Arm)

## Discussion

PREPARE-ABC is a pragmatic multi-centre randomised controlled trial aimed at investigating the clinical health benefits and cost-effectiveness of hospital-supervised and home-supported exercise programmes delivered as part of CRC care pathways within UK NHS Foundation Trusts. The trial has a hybrid effectiveness-implementation design, in which a parallel process evaluation component aims to explore the barriers and facilitators to implementation [15]. Based on these findings it appears that the interventions in PREPARE-ABC offer a clear and distinct approach to promoting exercise that was not observed in the delivery of standard care at any of the sites included in our sample. Although there was evidence that exercise within the context of daily activity was encouraged at some sites, this was typically limited to encouraging ongoing routine physical activity. During the pre-operative phase, advice, when given, was very general and focused on maintaining current activity levels. Similarly, post-operative advice was typically concerned with helping patients to return to their pre-operative levels of health and activity.

In our observations of standard care consultations, we did not identify staff attempting to provide support for exercise targets or motivating patients to exercise, and this probably reflects logistical challenges associated with providing exercise support within the resource-constrained NHS. The difficulty of engaging busy HCPs with many competing priorities in exercise provision cannot be overlooked [16]. Additionally, the short time period prior to CRC surgery presents a significant challenge for exercise prehabilitation. Targets set by NHS England dictate that following general practitioner referral for a suspected cancer, patients are to be investigated within 31 days and treated within 62 days, resulting in a time-window between decision to operate and surgery of 31 days (though this can vary due to medical decisions and availability of operating slots). Our process evaluation provides confirmatory evidence of these logistical challenges and suggests that hospitals need to implement systemic changes to effectively deliver pre- and post-operative exercise programmes with a motivational component. This will require planning to ensure pre-operative exercise is feasible given the short lead-in times to surgery and an assessment of capacity and resource allocation to provide dedicated exercise sessions within hospitals.

Staff successfully delivered each component of the hospital-supervised and home-supported exercise interventions, demonstrating a high level of fidelity to the intervention protocol. Patients also responded very positively to the exercise interventions, which suggests a good level of acceptability and potential effectiveness in scaled-up implementation. However, our findings clearly highlight the importance of HCPs tailoring the exercise counselling (motivational) aspects of the interventions according to patients’ exercise history and current level of motivation to increase activity levels. This evidence supports key tenets of SDT, specifically that the experience of patient autonomy and competence, in terms of previous exercise behaviours and self-efficacy, is critical for autonomous engagement which is ultimately associated with long term exercise participation [17, 18].

Participants also discussed the importance of socialisation and connection with the HCP (experiencing the need for relatedness), which is also associated with long-term behaviour change, in particular exercise uptake [18, 19]. This was evident for patients undertaking exercise sessions in the hospital-supervised arm as well as those receiving telephone counselling sessions in the home-supported exercise arm. In a similar way to the initial counselling sessions, HCPs need to flexibly deliver the supportive elements of these calls according to individual patient needs, thus supporting patient autonomy over their recovery and preventing exercise beyond their perceived capacity. However, the challenge lies in the extent to which HCPs can use the fundamental principles of motivational support to change patient behaviour in everyday cancer care settings and to consider which HCPs are best placed to do this; doctors, nurses, physiotherapists or other allied health care professionals. It is probable that hospitals will need to be pragmatic about which HCPs formally deliver the motivational sessions based on resources and availability, with constant re-enforcement with each HCP contact likely to be critical for improving effectiveness.

### Strengths and limitations

The findings from the process evaluation are limited by the available data, particularly the limited number of observations of intervention delivery. Delays in setting up sites for the main trial and process evaluation, compounded with a slow recruitment rate had an impact on our ability to purposively sample staff and patients and to obtain evidence from a range of appointments/sessions within each study arm. As a response, we focused on obtaining evidence across different data types within a more limited number of hospitals, enabling us to analyse data laterally to triangulate findings across staff and patient interviews, and observational fieldnotes. Future research should consider the relative risks and benefits of conducting process evaluation work during an internal pilot to optimise main trial implementation against later evaluation across a wider variation of participants and hospitals which might provide more in-depth insights into intervention delivery.

The sample included a high number of patients who reported already being motivated to exercise and carrying out a high level of exercise prior to participating in the trial. However, our main pilot study findings found that the majority of patients were only undertaking light intensity physical activity at the point of randomisation [14], suggesting that there was capacity for the intervention to motivate patients to increase activity levels. Our findings support this, the initial exercise counselling session provided a platform for nurturing autonomy and competency, while the ongoing social support sessions developed a sense of belonging, also deemed important for sustainable adherence to exercise interventions [18, 19]. The motivational component of exercise interventions embedded within cancer care pathways is therefore likely to be an important factor influencing the effectiveness of scaled-up NHS implementation beyond this research.

## Conclusion

This process evaluation provides evidence that hospital-supervised and home-supported exercise interventions delivered as part of the CRC care pathway are highly acceptable for many patients, and provide a motivational form of social support for helping to embed exercise in the patients’ preparation for surgical treatment and recovery. Appropriate communication skills are needed to ensure that motivational support is tailored to the individual, taking account of previous exercise behaviours and self-efficacy, to ensure patients feel adequately motivated and able to increase their activity levels. Delivery of hospital-supervised and home-supported exercise within the CRC pathway is feasible but systematic planning of capacity and resources is needed to optimise implementation. This may require significant changes to organisational and clinical delivery within colorectal units.

These findings generated the following recommendations which have since been incorporated into the main trial delivery:

1. Train staff on motivational skills and use of the intervention manual according to differing levels of expertise. This has now been incorporated into staff training to ensure that those delivering the intervention can follow the essential elements of the study protocol yet are able to use their existing skills to respond flexibly to different patients.
2. Trial team to work closely with sites early in trial set up to help ensure sufficient time to implement pre-operative exercise sessions prior to patients’ surgery, capacity to deliver supervised exercise within the hospital setting and allocation of dedicated staff to deliver the intervention.
3. Recommend that sites use a gel seat on exercise bikes following reported post-operative discomfort. Gel seats have been routinely provided to study sites in the main trial.

## Supplementary Information

**Additional file 1.** Site Profile Questionnaire.

**Additional file 2.** Standard Care Telephone Scoping Interview Guide.

**Additional file 3.** Deductive Coding Framework for Interviews.

## Data Availability

The datasets generated and/or analysed during the current study are not publicly available due to data transcripts including personal participant information not suitable for sharing, but are available from the corresponding author on reasonable request.

## Abbreviations

CNS: Colorectal nurse specialists
COREQ: Consolidated Criteria for Reporting Qualitative Research
CPET: Cardiopulmonary exercise test
CRC: Colorectal cancer
ERAS: Enhanced Recovery After Surgery
HCP: Healthcare professionals
NBOCA: National Bowel Cancer Audit
SDT: Self-determination theory

## References

[1] Cancer Research UK (CRUK). https://www.cancerresearchuk.org/health-professional/cancer-statistics/statistics-by-cancer-type/bowel-cancer#heading-Zero 2015.

[2] NBOCA (2019). National Bowel Cancer Audit.

[3] Lee CHA, Kong JC, Ismail H, Riedel B, Heriot A (2018). Systematic review and Meta-analysis of objective assessment of physical fitness in patients undergoing colorectal Cancer surgery. Dis Colon Rectum.

[4] West MA, Astin R, Moyses HE, Cave J, White D, Levett DZH, Bates A, Brown G, Grocott MPW, Jack S (2019). Exercise prehabilitation may lead to augmented tumor regression following neoadjuvant chemoradiotherapy in locally advanced rectal cancer. Acta Oncol.

[5] West MA, Lythgoe D, Barben CP, Noble L, Kemp GJ, Jack S, Grocott MPW (2014). Cardiopulmonary exercise variables are associated with postoperative morbidity after major colonic surgery: a prospective blinded observational study. Br J Anaesth.

[6] On behalf of the PREPARE-ABC Trial Collaborative. SupPoRtive Exercise Programmes for Accelerating REcovery after major ABdominal Cancer surgery trial (PREPARE-ABC): Study protocol for a multicentre randomized controlled trial. Colorectal Dis. 2021;00:1–11. 10.1111/codi.15805. 10.1111/codi.15805 34245094

[7] Deci EL, Ryan RM (1985). Intrinsic motivation and self-determination in human behavior.

[8] Physical activity guidelines: UK Chief Medical Officers’ report. A report from the Chief Medical Officers in the UK on the amount and type of physical activity people should be doing to improve their health. September 2019. Department of Health and Social Care, Llwodraeth Cymru Welsh Government, Department of Health Northern Ireland and the Scottish Government.

[9] Moore GF, Audrey S, Barker M, Bond L, Bonell C, Hardeman W, Moore L, O'Cathain A, Tinati T, Wight D, Baird J (2015). Process evaluation of complex interventions: Medical Research Council guidance. BMJ.

[10] Tong A, Sainsbury P, Craig J (2007). Consolidated criteria for reporting qualitative research (COREQ): a 32-item checklist for interviews and focus groups. Int J Qual Health Care.

[11] Braun V, Clarke V (2006). Using thematic analysis in psychology. Qual Res Psychol.

[12] Britten N, Pope C, Mays N (2006). Qualitative interviews in medical research. Qualitative research in health care.

[13] Geertz C, Martin M, McIntyre LC (1994). Thick description: toward an interpretive theory of culture. Readings in the philosophy of social science.

[14] On behalf of the PREPARE-ABC Trial Collaborative. SupPoRtive Exercise Programmes for Accelerating REcovery after major ABdominal Cancer surgery trial (PREPARE-ABC): Pilot phase of a multicentre randomised controlled trial. Colorectal Dis. 2021;00:1–15. 10.1111/codi.15856. 10.1111/codi.15856 34355484

[15] Landes SJ, McBain SA, Curran GM (2019). An introduction to effectiveness-implementation hybrid designs. Psychiatry Res.

[16] Kearney A, McKay A, Hickey H, Balabanova S, Marson AG, Gamble C, Williamson P (2014). Opening research sites in multicentre clinical trials within the UK: a detailed analysis of delays. BMJ Open.

[17] Teixeira PJ, Carraca EV, Markland D, Silva MN, Ryan RM (2012). Exercise, physical activity, and self-determination theory: a systematic review. Int J Behav Nutr Phys Act.

[18] Birtwistle SB, Ashcroft G, Murphy R, Gee I, Poole H, Watson PM (2019). Factors influencing patient uptake of an exercise referral scheme: a qualitative study. Health Educ Res.

[19] Ryan RM, Patrick H, Deci EL (2008). Facilitating health behaviour change and its maintenance: intervention based on self determination theory.

