# Supplementary material for: Implementing supportive exercise interventions in the colorectal cancer care pathway: a process evaluation of the PREPARE-ABC randomised controlled trial
Source: BMC Cancer. 2021 Oct 23;21:1137. doi: 10.1186/s12885-021-08880-8 (PMC8542291; doi:10.1186/s12885-021-08880-8)
Supplement: Supplementary file 1 — Additional file 1. Site Profile Questionnaire. [file 12885_2021_8880_MOESM1_ESM.docx]

##
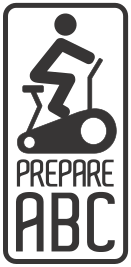
Supplementary File 1: Site Profile Questionnaire

- 1. How many new colorectal cancer cases were diagnosed in your hospital in 2016?
  2. How many resections does your unit carry out per year?
  3. How many beds are there on the colorectal surgery ward?
  4. Are your elective colorectal patients cohorted onto one ward?

If not, where are they managed?

- 1. How many staff work in the unit peri-operatively? (in this context peri- operative covers the period from admission day to discharge day)
  2. How many Clinical Nurse Specialists (CNS) are there as part of your unit?

| **Specialism** | **F/T** | **P/T** | **Vacancies?** |
| --- | --- | --- | --- |
| Colorectal |  |  |  |
| Stoma |  |  |  |
| Oncology |  |  |  |
| Enhanced Recovery |  |  |  |
| Other (specify) |  |  |  |

g. How many physios are there?

| **Type** | **F/T** | **P/T** | **Vacancies?** |
| --- | --- | --- | --- |
| ERAS Physio |  |  |  |
| Physio |  |  |  |
| Physio assistant |  |  |  |
| Other |  |  |  |
