# Supplementary material for: Implementing supportive exercise interventions in the colorectal cancer care pathway: a process evaluation of the PREPARE-ABC randomised controlled trial
Source: BMC Cancer. 2021 Oct 23;21:1137. doi: 10.1186/s12885-021-08880-8 (PMC8542291; doi:10.1186/s12885-021-08880-8)
Supplement: Supplementary file 2 — Additional file 2. Standard Care Telephone Scoping Interview Guide. [file 12885_2021_8880_MOESM2_ESM.docx]

Supplementary File 2: Standard Care Telephone Scoping Interview Guide

Telephone interview with Cancer Nurse Specialist at each unit.

PRE-OPERATIVE MANAGEMENT

1. Details of their patient pathway

a. How are patients informed of their diagnosis? *(Probes: When? With whom?)*

b. Do patients have a pre-operative assessment? *(Probes: When? With whom? i.e. nurse/ anaesthetist)*

c. Who sees them?

| **By Who?** | **Seen** | **Written info given** | **Outpatients or Pre-assessment or other?** |
| --- | --- | --- | --- |
| Colorectal Nurse |  |  |  |
| Stoma Nurse |  |  |  |
| Physio |  |  |  |
| Anaesthetist |  |  |  |
| Other (specify) |  |  |  |

e. If medical co-morbidities are identified pre-operatively how are they managed?

f. Are there any measures to medically optimise patients pre-operatively?

2. Details of current practice with regards to exercise

a. Are patients specifically offered any advice regarding exercise?

If yes, what advice is given and by whom?

b. Are patient’s fitness levels formally assessed pre-operatively? (CPET test?)

PERI-/ POST-OPERATIVE MANAGEMENT

1. Can you tell me about peri-operative management at your unit?

a. How are patients cared for on the day of admission?

b. Who are they seen by after the operation?

| **By Who?** | **How often?** |
| --- | --- |
| Colorectal Nurse |  |
| Surgical Nurse Practitioner |  |
| Enhanced Recovery Practitioner |  |
| Doctor |  |
| Physician’s Assistant |  |
| Physio |  |
| Other |  |

2. Explore the functioning of the unit

a. How many times are they reviewed by the clinical / nursing team?

b. How are ward rounds conducted?

c. Who leads the ward round (FY/ CT ST/ Consultant/ Nurse?)

d. Are patients seen daily?

3. Does the unit have an Enhanced Recovery Programme (ERAS) in place? (*Probes: Is it protocolised and the whole unit are involved or is it surgeon dependant?*)

- 1. If yes, then what are the details of delivery?

| **Phase** | **ERAS Item:** | **Y/N/ DK** |
| --- | --- | --- |
| Pre-operative | Pre-op counselling |  |
|  | Carbohydrate loading |  |
|  | No prolonged fasting |  |
|  | No/selective bowel prep |  |
|  | Antibiotic cover given |  |
|  | Thrombo prophylaxis given |  |
|  | No pre med |  |
|  | GP optimising medical conditions prior to referral |  |
| Intra-operative | Short acting anaesthetic |  |
|  | Mid-thoracic epidural |  |
|  | No drains |  |
|  | Intra operative warming |  |
| Post-Operative | No N/G tubes |  |
|  | Early catheter removal |  |
|  | Early oral nutrition |  |
|  | Non-opioid oral analgesia |  |
|  | Early mobilisation (on op day) |  |
|  | Stimulate gut motility (gum) |  |
|  | Do they have patient diaries? |  |

- 1. If no, then is there any other policy in place?

4. Patient stay

a. Are patients routinely sent to HDU / ITU post operatively?

b. How often are colorectal surgery patients seen by a physio post-operatively?

- Per day
- For how many days?
- Weekend physio?

c. Are outliers seen by the colorectal physio?

d. Do nurses mobilise patients outside of office hours?

POST-DISCHARGE

1. Post-discharge support

a. Do you have a discharge criteria to which patients are discharged? (Need to assess if meets trial criteria)

b. What post-operative support is offered to patients post-discharge by the unit?

| Type | How often/ when seen? | Face to face or remote? |
| --- | --- | --- |
| Nursing e.g. stoma nurse visit/ CNS support by phone |  |  |
| Medical e.g. remote clinic |  |  |
| Social |  |  |
| Other |  |  |

c. Do you have a cancer survivorship programme in place? If so, what does it look like / cover?

d. Do you have patient support groups in place? (*probe details*)

2. Details of current practice with regards to exercise

a. Are patients given any specific advice regarding exercise post-operatively?

If yes, what is it? Who gives it? In what format? Is it protocolised?

b. Are patients signposted to local exercise facilities?

GENERAL PERSPECTIVE

What is your general perspective on your department and how it has changed over a number of years?

DOCUMENTS: Are there any useful documents you are happy to share with the research team e.g. pathways; discharge flowchart; local policies; patient information, end of treatment summary template? (If they do not have a written document ask for a verbal run through using ours as a prompt)

Additional Comments/observations:
