# Supplementary material for: Implementing supportive exercise interventions in the colorectal cancer care pathway: a process evaluation of the PREPARE-ABC randomised controlled trial
Source: BMC Cancer. 2021 Oct 23;21:1137. doi: 10.1186/s12885-021-08880-8 (PMC8542291; doi:10.1186/s12885-021-08880-8)
Supplement: Supplementary file 3 — Additional file 3. Deductive Coding Framework for Interviews. [file 12885_2021_8880_MOESM3_ESM.docx]

**Supplementary File 3: Deductive Coding Framework for Interviews**

**Table 1: Deductive Coding Scheme (Patient Interviews)**

| **Headline Codes** | **Sub-Codes** |
| --- | --- |
| Activity Levels Pre-Trial | History of Exercise |
| Home Arm | Changes in exercise behaviour; Initial counselling session; Telephone calls |
| Hospital Arm | Changes in exercise behaviour; Initial counselling session; Supervised exercise sessions |
| Impact of trial | On self; On fitness; Unexpected outcomes |
| Logistics | Diary; Manual; Questionnaires; Pedometer; Therabands |
| Reason for taking part | Preference (group); Understanding of trial (including randomisation) |
| Research Staff | View of |
| Treatment Story | Complications; Decision Making; Diagnosis; Hospital Experience |
| Trial general experience | Expectations; Suggestions and improvements |
| Treatment As Usual | Change in Exercise behaviour |

**Table 2: Deductive Coding scheme (Staff Interviews)**

| **Headline Codes** | **Sub-Codes** |
| --- | --- |
| Challenges | Blinding; Recruitment; Logistical |
| Counselling Session (Initial) | How conducted; View of; Patient Response |
| HOME | How conducted (Telephone counselling); View of (Telephone counselling); Patient Response (Telephone counselling) |
| HOSP | How conducted (supervised exercise sessions); View of (supervised exercise sessions); Patient Response (exercise sessions) |
| Paperwork | Patient Diary; Patient Manual; Pedometer; Intervention Manual; Questionnaires; Therabands |
| Profession + Trial Role | Intervention Delivery; Recruitment |
| Training | View of |
| Trial – overall view | Expectations |
| TAU | Change in patient’s exercise behaviour |
| UEA Team | Process Evaluation |
